# Cost-effectiveness and equity impact of complex primary care interventions for disadvantaged populations

**DOI:** 10.3399/BJGPO.2025.0167

**Published:** 2026-03-25

**Authors:** Chloe Thomas, Ben Jackson, Caroline Mitchell, Josephine Reynolds, Daniel Hind

**Affiliations:** 1 Sheffield Centre for Health and Related Research (SCHARR), School of Medicine and Population Health, University of Sheffield, Sheffield, UK; 2 Faculty of Medicine and Health Sciences, Keele University, Keele, UK; 3 School of Healthcare, University of Leeds, Leeds, UK

**Keywords:** primary health care, general practice, inclusion health, disadvantaged, underserved, equity, health inequities, cost-effectiveness, distributional cost-effectiveness

## Abstract

**Background:**

Reducing health inequity is essential. The FAIRSTEPS (Framework to Address Inequities in pRimary care using STakEholder PerspectiveS) study developed and prioritised 28 vignettes describing complex primary care interventions targeted to disadvantaged groups, through Delphi consensus ranking by primary care practitioners for feasibility and perceived usefulness.

**Aim:**

To build on FAIRSTEPS by quantifying potential impacts of prioritised vignettes on cost-effectiveness and health equity.

**Design & setting:**

Simplified distributional cost-effectiveness analysis (DCEA) in England.

**Method:**

Pragmatic literature searches were carried out around each vignette to identify the following: (1) available economic evidence; and (2) information about size and distribution of populations targeted. Economic evidence was quality assessed using adapted National Institute for Health and Care Excellence (NICE) appraisal checklists. Extracted cost and quality-adjusted life-year (QALY) data and population data, were combined with published distributions of health opportunity costs and baseline lifetime health, to estimate net health benefits and equity measures for each vignette.

**Results:**

Suitable cost-effectiveness evidence was identified for 17 of 28 vignettes, with variable study quality and applicability. Fourteen vignettes were both cost-effective and equity-generating, with the most beneficial on both dimensions relating to community champions for health promotion; integrated care for people sleeping rough, engaged in sex work, or using drugs; and weight-loss programmes targeted at people on low incomes.

**Conclusion:**

Simplified DCEA using published data can be used to provide additional evidence to help prioritise complex primary care interventions aimed at disadvantaged populations, although the analysis is hindered by low quality economic data and limited study comparability. Further research estimating baseline health and health opportunity cost distributions across disadvantaged groups would improve accuracy of health equity assessments.

## How this fits in

The FAIRSTEPS (Framework to Address Inequities in pRimary care using STakEholder PerspectiveS) study identified 28 priority interventions aimed at reducing inequity across disadvantaged populations, which were considered to be both useful and feasible to implement within primary care settings. We extended FAIRSTEPS by carrying out simplified distributional cost-effectiveness analysis (DCEA), to quantify the cost-effectiveness and equity impact of FAIRSTEPS interventions enabling them to be ranked across four dimensions. We found that FAIRSTEPS prioritised interventions that are likely to be cost-effective and equity improving, providing reassurance to commissioners wishing to improve the health of their disadvantaged populations with limited primary care budgets. Together with the original FAIRSTEPS work, these findings offer practical guidance for practices, primary care networks, and commissioners, supporting the prioritisation of interventions that balance efficiency and equity with usefulness and feasibility.

## Introduction

Health equity remains a pressing issue in healthcare systems worldwide, with disadvantaged populations experiencing worse health outcomes. In England, socioeconomically deprived communities have lower life expectancy and poorer health compared with their more affluent counterparts.^1^ Disparities are driven by a complex interplay of structural determinants, including income, housing, education, and employment.^1,2^

Primary care plays a crucial role in addressing health equity, serving as the first point of contact for most patients and providing opportunities for preventive care. However, structural inequities in primary care provision persist, with general practices in deprived areas receiving fewer resources and having lower GP-to-patient ratios.^3^ Inclusion health populations such as migrants, people experiencing homelessness, and people engaged in sex work, with particularly poor health outcomes owing to multiple risk factors,^4,5^ face systemic barriers to registering with GPs, exacerbating disparities in care access.^6,7^

Despite decades of research and policy aimed at reducing inequity, progress has been inconsistent, and some interventions have inadvertently widened disparities.^8,9^ This phenomenon of intervention-generated inequalities is observed in interventions requiring high levels of individual engagement, such as diabetes education or cancer screening.^10–13^ Conversely, structural and fiscal interventions, for example, tobacco taxation or provision of resources, are more likely to reduce inequities.^14^ Given these complexities, there is an urgent need for decision makers to prioritise interventions that both improve health outcomes and reduce disparities.

Economic evaluations are key in shaping healthcare policy by assessing intervention cost-effectiveness. However, traditional cost-effectiveness analysis prioritises maximising total health gains, neglecting distributional impacts, and failing to consider whether interventions disproportionately benefit advantaged groups.^15^ DCEA provides a structured method for evaluating both cost-effectiveness and equity impacts of interventions, by quantifying health gains across different demographic groups.^16^ Despite its potential, policy application of DCEA has been limited owing to data constraints and lack of standardised methods for integrating equity weights into economic evaluations.^17,18^

The FAIRSTEPS study developed an evidence-informed framework for designing, implementing, and evaluating localised health equity interventions in primary care, and a set of prioritised exemplar complex intervention ‘vignettes’.^19^ Literature searches were conducted to identify studies describing primary care interventions designed to address inequity. Varied study types described processes, barriers, and occasionally outcomes. Thematic analysis enabled key elements to be identified and synthesised into vignettes, each summarising an intervention and target population for that intervention (Table 1).^19^ A Delphi consensus study combining primary healthcare practitioner and public perspectives enabled ranking of vignettes by ease of implementation and usefulness (how effective they were thought to be for reducing health disparities). This enabled a priority set of intervention examples to be identified, giving primary care decision makers a starting place for reducing inequity along with a framework to help adapt or develop new interventions for local contexts.^19^

**Table 1.** Summary description of each vignette (full descriptions can be found in Supplementary Tables S3 and S4). No economic data were found to inform vignettes in italics (11/28 vignettes).

ID	Vignette description
*1a*	*Staff education to increase knowledge around equity-oriented care for people in socially deprived and marginalised populations.*
*1b*	*Online e-training package for primary care practitioners to support understanding of issues relating to primary care for refugees and asylum-seekers.*
*1c*	*Training programme for primary care teams on supporting the health of people with learning difficulties.*
*1e*	*3-year training programme for GP specialty trainees rotating through practices with high levels of socioeconomic deprivation.*
*1f*	*One-off GP training placements with charity and community groups to enable learning around care for refugees, asylum-seekers, and other migrants.*
*1g*	*A community-based placement for medical students focused on health care for people with disabilities.*
1h	Multidisciplinary team meetings and extended GP consultations and case review for patients with complex needs.
*2b*	*Integration of adults experiencing homelessness into mainstream healthcare centres by registering patients with general practices.*
2e	Promotion of uptake for cervical screening in people whose first language is not English.
2f	Extended consultations for refugees and asylum-seekers, with supporting resources and signposting to mental health support.
2h	Weekly group weight-loss programme (diet and exercise) targeted at people with low incomes.
2j	Easy referral pathways to targeted mental health support for vulnerable individuals (for example, experiencing homelessness, socially isolated, adolescent).
2l	Trained advisers to give welfare rights advice and assistance with benefit entitlements for patients and their carers.
2m	Group community sessions to educate women on cancer screening and prevention.
*2n*	*Systematic flagging of transgender patients during cancer screening recall to ensure that future recall targets patients who require it.*
*2q*	*A ‘safe surgery’ programme for migrants in vulnerable situations, including poster information and practice training.*
*3a*	*Wellbeing diaries and/or handheld health records for adolescents or adults with learning difficulties.*
3c	A healthcare service for people sleeping rough, people engaged in sex work, and/or vulnerable migrants, with additional support for basic needs.
3d	A special primary care centre to deliver integrated care for at-risk young people, people engaged in sex work, and people using intravenous drugs.
3e	Intensive case management for people experiencing homelessness and people on low income, who may also have mental health problems or lack social support.
*3g*	*Additional health screening for new refugees and asylum-seekers to provide links with primary health care, community, and settlement support.*
3i	Socially tailored group health coaching using behavioural change approaches to improve CVD risk factors and support chronic conditions.
3m	Case finding healthcare van driving to areas where people experiencing homelessness live, with mobile testing equipment for TB and other conditions.
3n	Targeted support for domestic violence victims and their families from trained health and wellbeing workers.
4a	Programme supporting access for healthcare appointments for those with transport difficulties.
4b	Community or charity-led buddying service to help people with difficulties accessing care support in making and attending appointments.
4c	A targeted service across a group of practices to increase access for older patients from deprived groups providing transport for appointments.
4f	Local community health champions working with community groups to improve health.

CVD = cardiovascular disease. TB = tuberculosis

This analysis builds on the FAIRSTEPS work described above by incorporating DCEA based on existing published evidence to determine the cost-effectiveness and equity impacts of the prioritised FAIRSTEPS vignettes.

## Method

DCEA requires data about expected costs and health benefits of interventions, and the likely distribution of those costs and benefits in the population. Existing published evidence was sought to inform this analysis. This project was conducted with limited resources, meaning it was not feasible to conduct systematic reviews of the relevant evidence for all 28 prioritised FAIRSTEPS vignettes. A simplified, pragmatic search and review strategy was therefore developed with the aim of identifying published data informing either the value or distribution of costs and benefits for each vignette. A summary is given below with more detailed information in supplementary material.

### Economic data

Searches were performed to identify published estimates of costs and health benefits (quality-adjusted life years; QALYs) for each of the 28 vignettes described in the FAIRSTEPS study (Table 1).^19^ Search terms were developed based around each described intervention and population (Supplementary Table S1) and combined to enable a set of related searches to be carried out for each vignette. To enable rapid identification of results encompassing both academic papers and grey literature, searches were undertaken via Google Scholar and Google, and titles and summaries of search results examined for relevancy until no further relevant results were identified.

Studies were selected for full-text review if economic outcomes were indicated, and if they evaluated an intervention that matched vignette description. At full-text review, a single study was prioritised to inform each vignette analysis, or a small number of complementary studies covering different aspects of multi-component interventions. Studies were excluded if the intervention evaluated was too dissimilar to the vignette, or if separate cost and QALY information was absent. Remaining studies were prioritised based on the following: similarity of intervention to vignette; similarity of target population to vignette; study setting; quality of evidence (study design, study size, study duration, publication source); and study year. Supplementary Table S1 shows all studies reviewed at full text for each vignette and reasons for inclusion and exclusion.

For each identified study, key characteristics were extracted (Supplementary Table S2), plus cost and QALY outcome data. Applicability and quality assessment was carried out based on National Institute for Health and Care Excellence (NICE) guidelines appraisal checklists,^20^ modified to increase relevance to the study (Supplementary Table S3). Extracted cost and QALY data were scaled per person. Costs from non-UK studies were converted to GBP and all costs were inflated to 2022–2023 values.^21,22^

### Population data

It was assumed that the distribution of health benefits for each intervention would be equivalent to the target population distribution. Grey literature searches were undertaken for information relating to size and distribution (by sex and quintiles of socioeconomic deprivation) in England of populations targeted by each vignette. Information from high quality sources, such as national statistics, was prioritised where available. Population groups were defined based on vignette wording and refined where necessary to align directly with definitions in identified data sources. Population numbers were extracted and scaled to the estimated 2023 population size for the specified age group in England.

### Simplified DCEA

DCEA was performed using the method described in Griffin *et al* (2019).^23^ Net health benefit (a measure of cost-effectiveness) was estimated for each vignette across the whole population and in subgroups specified by sex and deprivation quintiles, using the extracted economic and population data. Estimating the equity impact of each vignette additionally required data about quality-adjusted life expectancy (QALE) for each sex and deprivation subgroup, which has previously been estimated for England.^24^ An adjusted distribution of QALE was estimated for each vignette by adding subgroup net health benefit for that intervention to lifetime health. Equity measurements (such as the slope index of inequality) were calculated with and without intervention and the change in equity quantified. Cost-effectiveness and equity were also combined into a single measure called Equally Distributed Equivalent (EDE) health, which represents a revised estimate of cost-effectiveness if public preferences for equity are taken into account. A full technical description of what these measures mean and how DCEA was carried out is available in supplementary material.

## Results

Seventy-six potentially useful economic evaluations were identified through title and abstract searches (Supplementary Table S1), which were narrowed down using inclusion and exclusion criteria at full-text review to 17 studies, covering 17 of the 28 vignettes, with five studies informing more than one vignette and three vignettes covered by more than one study (Supplementary Table S2). No study was found fulfilling inclusion and exclusion criteria for 11 vignettes (Supplementary Table S4); six of these related to training of medical students or practice staff, with others relating to interventions targeted at refugees, migrants or asylum-seekers, people with disabilities or learning difficulties, and LGBTQ+ or transgender patients.

Applicability and quality varied widely between selected studies (Table 2). Applicability scores were highest for reporting of incremental costs and QALYs, and poorest for all important and relevant outcomes being represented in the cost and QALY outcomes, often owing to short study durations that ignored potential longer-term outcomes (Supplementary Table S5). For quality, the highest scoring domain related to taking a health perspective, but scores were poorest around how health benefits were estimated, with many studies using non-standard methods (Supplementary Table S6).

**Table 2.** Summary outcome data from the cost-effectiveness studies used to inform each vignette, including averaged applicability and quality scores, and extracted and processed cost and QALY data

ID	Study (first author and year)	Average applicability score^a^	Average quality score	Study costs per person (country if not in UK £)	Study cost year	Study inflated converted costs per person (£)^b^	Study QALYs per person	Vignette costs per person (£)	Vignette QALYs per person
1h	Mercer *et al*. 2016^^30^^	4.4	4.4	£929	2012	£1167.83	0.0760	£1167.83	0.0760
2f	Mercer *et a* 2016^^30^^	3.7	4.4	£929	2012	£1167.83	0.0760	£1167.83	0.0760
2j	Balmer and Pleasence 2012^^31^^	2.6	2.1	£36.45	2011–2012	£45.82	0.0149	£45.82	0.0149
2l	Howel *et al* 2019 ^^32^^	3.6	3.4	£43.76	2013–2014	£53.49	0.0090	£53.49	0.0090
2e	Tsiachristas *et al* 2017^^28^^	3.7	4.3	£4.34	2014	£5.26	0.0005	£5.26	0.0005
2m	Tsiachristas *et al* 2018 ^^28^^	3.0	4.3	£4.34	2014	£5.26	0.0005	£56.00^c^	0.0007^c^
	Anderson *et al* 2002^33^	2.7	2.8	$30.45 (US)	1995	£45.60	NA		
	Pharoah *et al* 2013^34^	3.0	4.0	£3.89	2009	£5.14	0.0002		
2h	Ahern *et al* 2022 ^^35^^	4.4	4.8	-£424^d^	2018–2019	-£487.27	0.0298^d^	-£487.27	0.0298
3i	Ahern *et al* 2022 ^^35^^	3.6	4.8	-£336^d^	2018–2019	-£386.14	0.0248^d^	-£386.14	0.0248
3c	Collins *et al* 2013^^36^^	3.0	3.0	£101.84	2012–2013	£125.89	0.0112	£125.89	0.0112
3d	Collins *et al* 2013^^36^^	3.1	3.0	£102	2012–2013	£125.89	0.0112	£1114.89^c^	0.4185^c^
	Sweeney *et al* 2019^^37^^	3.9	4.2	-£564	2013–2014	-£689.71	0.1973		
	Brogan *et al* 2019^38^	3.6	4.0	£1492	2017	£1678.71	0.2100		
3e	Parsonage *et al* 2014 ^^39^^	3.4	3.0	£885	2012–2013	£1093.93	0.0810	£1093.93	0.0810
3m	White *et al* 2011^^40^^	4.1	4.1	-£920	2009	-£1216.36	0.0829	£949.85^c^	0.1075^c^
	Ward *et al* 2019 ^^41^^	4.0	4.6	£232	2018	£266.51	0.0247		
3n	Barbosa *et al* 2018 ^^42^^	4.1	4.2	£59.78	2015–2016	£72.15	0.0152	£72.15	0.0152
4a	Ford *et al* 2019 ^^25^^	3.6	3.8	£17.01	2016–2017	£20.11	-0.1700	£20.11	-0.1700
4c	Ford *et al* 2019^^25^^	3.1	3.8	£17.01	2016–2017	£20.11	-0.1700	£20.11	-0.1700
4b	Tarride *et al* 2024^^43^^	3.1	3.0	-$114 (Canada)	2021	-£61.01	0.0012	-£61.01	0.0012
4f	Visram *et al* 2020^^44^^	2.7	2.1	£352.93	2014–2015	£427.62	0.0905	£427.62	0.0905

^a^Applicability score reflects applicability to the specified vignette, so will differ where the same study is used for more than one vignette. ^b^Currency conversion is based on purchasing power parities for the study cost year. All costs are inflated to 2022–2023 values using the NHS pay and prices cost inflation index. ^c^Costs and QALYs are summed where multiple studies have been used to inform a single vignette. ^d^Different intervention duration used to inform costs and QALYs for these two vignettes that use outcome data from the same study. NA = not applicable, study only used for intervention cost. QALY = quality-adjusted life year.

Population size and distribution was estimated for all vignettes (Supplementary Table S7 and Supplementary Table S8). Estimated population sizes in England ranged from 37 845 for people sleeping rough to 19 192 318 for people from socioeconomically deprived or ethnic minority communities. While vignettes varied in estimated distribution by sex, all estimated distributions skewed towards the most deprived end of the socioeconomic scale.

**Figure 1.**
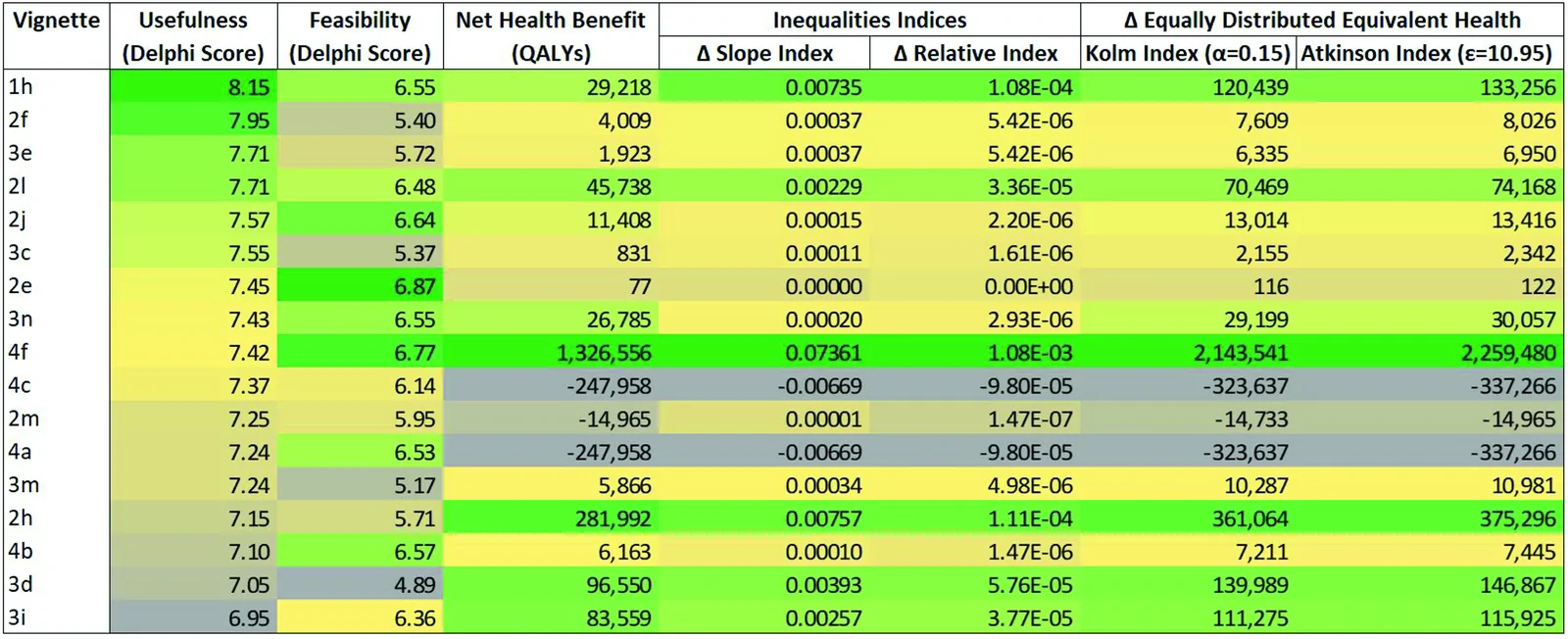
Multi-way colour-coded ranking of vignettes for usefulness and feasibility (median Delphi scoring out of 10 from Jackson *et al*),^19^ cost-effectiveness (using net health benefit measured in QALYs) and equity (measured using the change in the slope index of inequality and the change in the relative index of inequality) across the English population. The change in equally distributed equivalent health combines cost-effectiveness and equity into a single indicator using either the Kolm index (absolute inequality) or the Atkinson Index (relative inequality), based on elicited inequality aversion parameters.^45^ Negative values represent vignettes that are not cost-effective and/or reduce equity. Green shading represents the most beneficial and grey shading the least beneficial in each domain. QALYs = quality-adjusted life years

Based on extracted economic data, 15 interventions resulted in QALY gain and three were cost-saving (Table 2). DCEA estimates of net health benefit suggested 14 vignettes would be cost-effective (Figure 1) with the most cost-effective at the English population level relating to 4f: community health champions, and 2h: weight loss programmes for people with low income, primarily owing to the large size of the targeted population. If net health benefit was instead estimated per person targeted (Supplementary Figure S1), cost-effectiveness rankings changed slightly with integrated care for inclusion health groups such as people experiencing homelessness, people engaged in sex work, or people using intravenous drugs being most cost-effective (3d & 3m).

**Figure 2.**
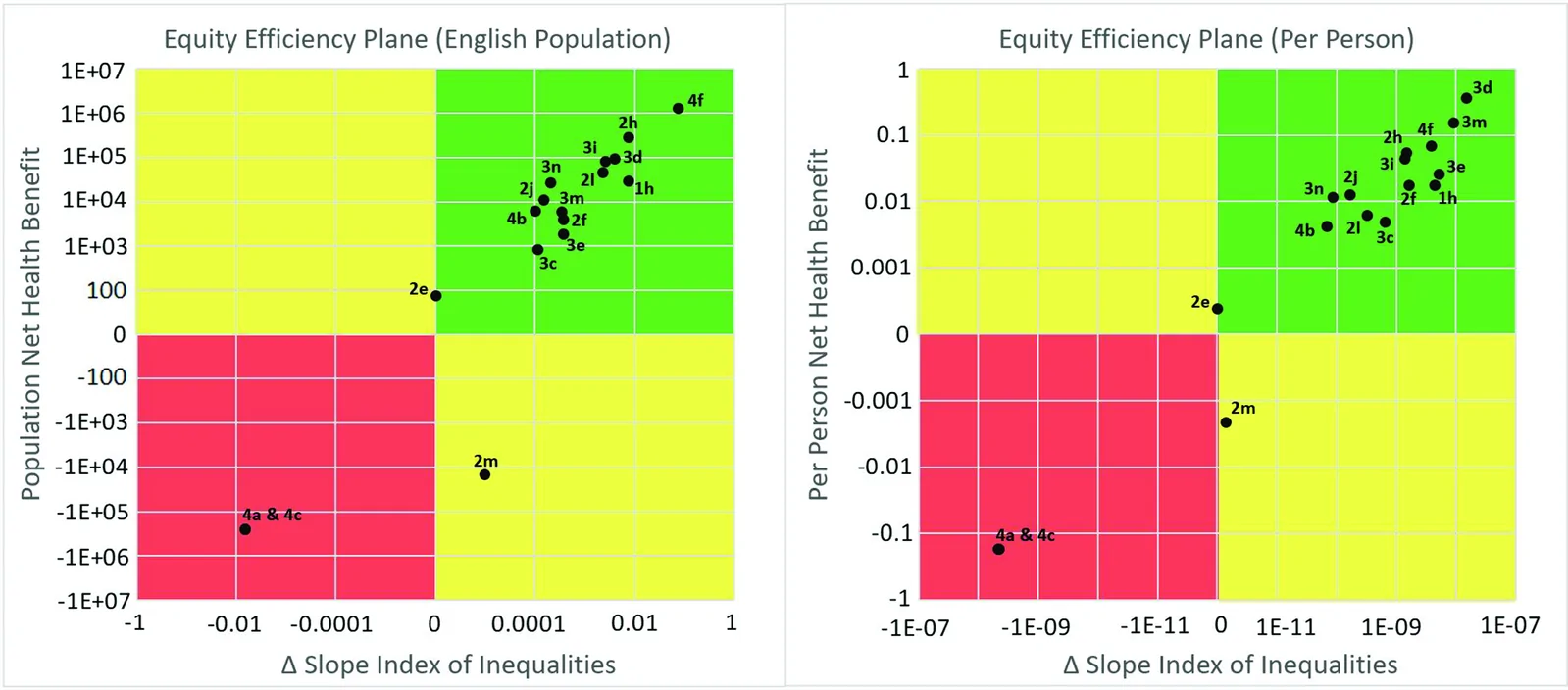
Vignettes ranked on the equity-efficiency plane if either the entire eligible English population is targeted, or if only a single eligible person is targeted to receive the intervention described in each vignette. In the latter plane, the size of the population does not impact the positioning of each intervention on the plane. The green shading (top right quadrant) represents a win-win scenario, where interventions are both equitable and cost-effective, whereas red shading (bottom left quadrant) represents a lose-lose scenario where interventions are neither equitable nor cost-effective. Net health benefit is measured in quality-adjusted life years (QALYs). Axes are subject to a log transformation to enable all vignettes to be displayed on a single plane

Most cost-effective interventions were also equitable and vice versa, as shown by their location in the win-win quadrant of the equity-efficiency plane (Figure 2). Ranking of vignettes was similar against measures of relative or absolute equity (Figure 1 & Supplementary Figure S1). In 15 vignettes, estimated EDE impacts were greater than estimated net health benefit indicating that cost-effectiveness would be increased if equity preferences were taken into account. Two vignettes (4a and 4c), informed through the same cost-effectiveness study,^25^ were estimated to be neither cost-effective nor equity generating owing to reductions in health-related quality-of-life observed in the intervention group. However, the study was a small feasibility trial not powered for effectiveness. Promotion of cancer screening in women at high risk (2m) was estimated as equitable but not cost-effective, owing to marginal cost-effectiveness of breast cancer screening and high intervention cost.

Some vignettes performed strongly in all four dimensions (useful, feasible, cost-effective, equitable). The most useful intervention (1h: multidisciplinary care for patients with complex needs) was also third of all ranked interventions for feasibility, third for equity impact, and sixth for cost-effectiveness. In contrast, the most feasible intervention for which cost-effectiveness evidence was identified (2e: promoting uptake of cervical screening in people whose first language is not English) was only marginally cost-effective and equitable.

## Discussion

### Summary

FAIRSTEPS identified and prioritised complex primary care interventions that consensus suggested would be useful and easy to implement in services for disadvantaged populations, despite the lack of evaluation data.^19^ This study extends FAIRSTEPS by estimating impact on cost-effectiveness and equity of these interventions using simplified DCEA based on published literature. The analysis confirms that FAIRSTEPS-prioritised interventions with relevant economic data frequently align with the ‘win-win’ quadrant of the equity–efficiency plane, meaning they improve health outcomes efficiently while reducing inequity. The relative placing of different interventions on the plane is influenced by both the magnitude of per person impacts and target population size. This illustrates that equity improvements may be achieved either through marginal health improvement in a large moderately deprived group, or by targeting a small number of highly disadvantaged people with high-impact interventions.

### Strengths and limitations

This work expands FAIRSTEPS’ utility beyond expert consensus on feasibility and usefulness to include structured economic evaluation, providing essential additional information for decision makers given limited primary care budgets. Use of DCEA is still uncommon, with this study being first to use it across multiple complex primary care interventions for which evidence is sparse and heterogeneous. The aggregation of economic and population data also represents a useful resource for research and policy. The analysis also highlighted areas where cost-effectiveness data are missing, which could be targets for future research priorities.

Several limitations must be acknowledged. The study relied on a simplified DCEA approach,^23^ incorporating data from existing published economic evaluations rather than conducting new cost-effectiveness studies. Furthermore, owing to resource constraints, it was not possible to conduct systematic literature searches across multiple databases or robust synthesis of identified data for all 28 vignettes. This means relevant studies may have been missed, and the data incorporated in DCEA may not have been optimally representative of the cost-effectiveness of each vignette. Identified economic studies were highly heterogeneous in study design and quality, limiting comparability across vignettes, and the lack of economic data to inform 11 vignettes limits the generalisability of the findings. Additionally, while the analysis accounts for health inequities by sex and deprivation, it does not fully capture potential equity benefits for highly marginalised subpopulations, such as individuals experiencing homelessness, asylum-seekers, or people with disabilities, whose baseline health is considerably poorer.^5^ This means that while the general findings of cost-effectiveness and improved equity are likely to hold true, caution must be exercised around the relative ranking of interventions.

### Comparison with existing literature

Prior studies have highlighted the importance of integrating equity into economic evaluations, although few have focused on complex primary care interventions. Economic modelling of a Brazilian community health workers programme highlighted the complexities of evaluating complex system interventions, given greater data requirements of DCEA compared with standard economic analysis.^26^ In the UK, DCEA has been used to evaluate cost-effectiveness and equity impacts of primary care interventions such as screening and brief intervention for alcohol,^27^ and cervical cancer screening uptake interventions.^28^ These studies involved bespoke model development, which is impractical when many diverse interventions must be assessed. Previous work has demonstrated that simplified DCEA based on pre-existing primary analysis is a feasible method for assessing large numbers of interventions.^23,29^ The work presented here indicates that similar methods can help provide reassurance that locally generated interventions are likely to be in the win-win quadrant, before committing to long-term funding.

### Implications for research and practice

Together with the original FAIRSTEPS work, these findings offer practical guidance for practices, primary care networks, and commissioners, supporting the prioritisation of interventions that balance efficiency and equity with usefulness and feasibility, and providing reassurance that similar interventions are likely to be good value for money. Certain interventions rank highly on multiple dimensions, indicating potential for easily implementable, cost-effective reduction of health inequities. This study demonstrates how integrating DCEA into decision making enhances the prioritisation of equity-sensitive primary care interventions, bridging the gap between expert consensus and economic evaluation.

One area for future research is identifying which FAIRSTEPS interventions require further economic evaluation. Evidence was missing for some vignettes and for others data were of low quality, subject to insufficient sample sizes, or omitting potential longer-term costs and benefits. The complexity of these interventions makes evaluation challenging and the benefits of intervention may vary widely depending on how they are implemented, the population targeted, and local contexts. Clarifying which interventions require additional economic modelling and developing methods to do this while taking complexities into account would strengthen the applicability of this approach. Additionally, further refinement of DCEA methods would enable inclusion of more granular equity impacts beyond deprivation and sex. Future work should aim to explore inclusion groups and intersectional factors to ensure existing inequities and equity benefits of intervention are fully captured.

## Data

All data generated by this project is provided either in the main manuscript or the supplementary tables and figures.
